# Ultrasonographic findings in female inmates in a prison unit in the state of São Paulo, Brazil

**DOI:** 10.1590/0100-3984.2024.0086

**Published:** 2024-12-02

**Authors:** Soraya Gomes de Amorim Andrade, Fernando Moreira de Andrade, Edward Araujo Júnior, Wagner José Gonçalves, José Mendes Aldrighi

**Affiliations:** 1 Faculdade de Saúde Pública da Universidade de São Paulo (FSP-USP), São Paulo, SP, Brazil; 2 Escola Paulista de Medicina da Universidade Federal de São Paulo (EPM-Unifesp), São Paulo, SP, Brazil; 3 Universidade Municipal de São Caetano do Sul (USCS), São Caetano do Sul, SP, Brazil

**Keywords:** Prisons, Women, Prisoners, Ultrasonography, Mass screening., Prisões, Mulheres, Prisioneiros, Ultrassonografia, Programas de rastreamento.

## Abstract

**Objective:**

To characterize the ultrasonographic findings in female inmates in a prison unit in the state of São Paulo, Brazil, and to analyze the associations between ultrasonographic findings and sociodemographic characteristics.

**Materials and Methods:**

This was a retrospective cohort study that analyzed the ultrasonographic examinations performed in consecutive female inmates in a prison unit in the city of São Paulo, between 2015 and 2020. The following ultrasound examinations were performed: soft tissue, thyroid, cervical, breast, transvaginal, pelvic gynecology, total abdomen, upper abdomen, and kidney/urinary tract in B-mode, with color Doppler, or both.

**Results:**

The sample consisted of 478 women who underwent 1,274 ultrasound examinations. The mean age was 40.0 years (range, 22-73 years). Over half (58.2%) of the women were single, 50.2% were White, and 59.6% self-identified as heterosexual. An ultrasonographic finding of uterine myoma was associated with older age (*p* = 0.022), higher body mass index (*p* = 0.022), and being tattooed (*p* = 0.040). An ultrasonographic finding of simple ovarian cyst was associated with sexual orientation (*p* = 0.020), whereas a finding of adenomyosis were associated with older age (*p* = 0.012). An ultrasonographic finding of polycystic ovaries was associated with younger age (*p* < 0.001). The most common ultrasonographic findings were uterine myoma (in 13.6%), biliary lithiasis (in 13.4%), and renal lithiasis (in 11.5%).

**Conclusion:**

The most common ultrasonographic findings in female inmates were uterine myoma, biliary lithiasis, and renal lithiasis.

## Abstract (pt)

**Objetivo:**

Caracterizar os achados ultrassonográficos de mulheres detentas em uma unidade prisional do Estado de São Paulo e analisar a associação entre os achados ultrassonográficos e as características sociodemográficas.

**Materiais e Métodos:**

Trata-se de um estudo consecutivo retrospectivo tipo coorte que analisou os exames ultrassonográficos de detentas de uma unidade prisional da cidade de São Paulo, entre 2015 e 2020. Foram realizados os seguintes exames ultrassonográficos: tecidos moles, tireoide, cervical, mama, transvaginal, ginecológico pélvico, abdome total, abdome superior, e rim e vias urinárias em modo-B e/ou Doppler colorido.

**Resultados:**

A amostra foi composta por 478 mulheres que realizaram 1.274 exames de ultrassonografia. A média de idade foi 40,0 anos, variando de 22 a 73 anos. Pouco mais da metade das mulheres eram solteiras (58,2%), brancas (50,2%) e se autoidentificaram como heterossexuais (59,6%). O achado ultrassonográfico de mioma uterino foi associado a idade mais avançada (*p* = 0,022), maior índice de massa corporal (*p* = 0,022) e tatuagens (*p* = 0,040). O achado ultrassonográfico de cisto ovariano simples foi associado a orientação sexual (*p* = 0,020), enquanto a adenomiose foi associada a idade mais avançada (*p* = 0,012). Os achados ultrassonográficos de ovários micropolicísticos foram associados a idade mais jovem (*p* < 0,001). Os achados ultrassonográficos mais comuns foram mioma uterino (13,6%), litíase biliar (13,4%) e litíase renal (11,5%).

**Conclusão:**

Os achados ultrassonográficos mais comuns em mulheres detentas foram mioma uterino, litíase biliar e litíase renal.

## INTRODUCTION

Currently, the Brazilian prison system houses a total of 663,387 people of both sexes deprived of their liberty, with those in the state of São Paulo accounting for 30.17%. There are 200,178 inmates imprisoned in the state of São Paulo, of whom 8,897 are women, representing 4.44% of all prisoners in the state and 30.92% of all female inmates in the country as a whole^(1)^. The confinement experienced by female inmates and the resulting lack of contact with family members creates expectations that result in a continuous process of physical and emotional stress that can lead to physical and mental illness^(2,3)^. Therefore, early detection of health problems is particularly important, because there are few strategies to control chronic diseases within the prison system^(4,5)^.

The population of female inmates in Brazil has certain characteristics. Most such inmates are young and typically have unprotected sex, as well as sex with multiple partners, thereby increasing their risk of sexually transmitted diseases, such as infection with human papillomavirus, which is associated with cervical cancer^(6,7)^. Women in prison have little access to tests for early detection, the most common being preventive cytology for cervical cancer by Pap smear^(8)^.

Women who have been incarcerated are significantly less likely to receive adequate prenatal care, including ultrasound examination, than are women in the general population^(9)^. Female inmates rarely undergo imaging examinations to detect problems at an early stage, particularly in areas at risk for cancer, such as the breasts, lungs, thyroid, abdomen, and pelvic region. Because ultrasound is a simple imaging modality that is free of ionizing radiation, inexpensive, and accessible, it could be an appropriate method for screening female prisoners for disease.

To our knowledge, there have been no studies evaluating ultrasonographic findings in female inmates. The aim of this study was to characterize the ultrasonographic findings of female inmates in a prison unit in the state of São Paulo, describing the most common conditions, as well as to analyze the association between ultrasonographic findings and sociodemographic characteristics.

## MATERIALS AND METHODS

### Type of study

This was a retrospective cohort study that analyzed ultrasound examinations conducted between 2015 and 2020 of consecutive female inmates from a prison unit in the city of São Paulo. All ultrasound examinations were performed at the Cruz de Malta Treatment Center, based on a partnership between the prison unit and this nonprofit health care facility. All ultrasound examinations, as well as the collection of clinical and demographic data, were performed by an examiner who had 24 years of experience in general ultrasound and was board certified by the Brazilian College of Radiology. The study was approved by the Research Ethics Committee of the University of São Paulo (Reference no. 69572723.0.0000.5421).

### Ultrasound examination

Ultrasound examinations were performed with a portable ultrasound system (Logiq P5; GE HealthCare, Milwaukee, WI, USA) equipped with linear, convex, and endocavity transducers (11L, 5C, and E8C, respectively; GE HealthCare). The following ultrasound examinations were performed: soft tissue, thyroid, cervical, breasts, transvaginal, pelvic gynecology, total abdomen, upper abdomen, kidneys, and urinary tract in B-mode, with color Doppler, or both. The scheduling of ultrasound examinations varied according to the clinical indications, and more than one ultrasound examination could be performed in the same woman. Preparation for the ultrasound examinations varied according to the type of examination. Women who declined to undergo the ultrasound examination, pregnant women, and women with emergency conditions were excluded.

### Sociodemographic data

The following sociodemographic variables were assessed: age, nationality, education level, marital status, body mass index (BMI), ethnicity, profession, religion, sexual orientation, and the presence of tattoos. Female inmates remain in the semi-open prison system for up to three years.

### Statistical analysis

Data were transferred to a Microsoft Excel spreadsheet, and statistical analyses were performed with the IBM SPSS Statistics software package, version 24.0 (IBM Corp., Armonk, NY, USA) and R software version 3.6.3 (The R Project for Statistical Computing, Vienna, Austria). Statistical analysis was performed using summary measurements such as mean, median, minimum/maximum values, standard deviation, absolute values, relative frequencies (percentages), including the creation of pie charts, bar graphs, box plots, and one-dimensional scatter plots. The inferential analyses used to confirm or refute the evidence found in the descriptive analysis were the Mann-Whitney U test, Pearson’s chi-square test, and Fisher’s exact test or its extension. A 5% alpha significance level was used for all inferential conclusions.

## RESULTS

The initial sample comprised 489 women. However, 11 women were excluded: three because they declined to undergo the ultrasound examination; five because they were pregnant; and three because they presented with emergency conditions. Therefore, the final sample comprised 478 women who underwent a total of 1,274 ultrasound examinations. The majority of the women were Brazilian (93.1%), followed by Peruvian (0.8%), Bolivian (0.6%), and South African (0.6%), and 89.7% had some type of tattoo. As shown in Table 1, the mean age was 40.0 years (range, 22-73 years). Just over half of these women were single (58.2%), white (50.2%), and self-identified as heterosexual (59.6%). Only 49 (10.3%) of the women had completed 9 years of schooling and 101 (21.1%) had completed 12 years of schooling. The most common religion among this group of women was evangelical (44.8%), followed by catholic (38.9%). The most common occupations were homemaker (in 18.4%), saleswoman (in 7.3%), domestic worker (in 6.9%), and hairdresser (in 5.4%). The mean body weight was 66.4 kg (range, 41-135 kg), the mean height was 1.62 m (range, 1.45-1.80 m), and the mean BMI was 25.22 kg/m^2^ (range, 16.53-49.59 kg/m^2^).

**Table 1:** Sociodemographic characteristics of the female inmates (N = 478).

Sociodemographic characteristic	Result
Age (years)	
Mean ± SD	40.0 ± 10.5
Median (range)	39 (22-73)
Level of education, n (%)	
None	14 (1.5)
< 9 years of schooling	209 (43.7)
9 years of schooling	49 (10.3)
High school, incomplete	83 (17.4)
High school, complete	101 (21.1)
College, incomplete	14 (2.9)
College, complete	8 (1.7)
Marital status, n (%)	
Common-law marriage	106 (22.2)
Married	43 (9.0)
Divorced	26 (5.4)
Separated	10 (2.1)
Single	278 (58.2)
Widowed	15 (3.1)
Ethnicity, n (%)	
Asian	1 (0.2)
White	240 (50.2)
Black	60 (12.6)
Mixed	177 (37.0)
Tattooed, n (%)	
No	49 (10.3)
Yes	429 (89.7)
Religion	
Catholicism	186 (38.9)
Christianity	35 (7.3)
Spiritism	39 (8.2)
Evangelical Christianity	214 (44.8)
Islam	2 (0.4)
None	2 (0.4)
Sexual orientation, n (%)	
Bisexual	106 (22.2)
Heterosexual	285 (59.6)
Homosexual	87 (18.2)

All 478 women underwent multiple ultrasound examinations: upper and total abdomen (in 70.1%); transvaginal (in 65.3%); gynecological pelvic (in 48.3%); breast (in 36.6%); Doppler flow study (in 14.0%); soft tissue (in 11.3%); thyroid (in 9.6%); and kidneys/urinary tract (in 7.1%). It is worth noting that some women underwent more than one examination during the study period.

The ultrasound examinations revealed several important findings, the most common of which were uterine myoma, biliary lithiasis, renal lithiasis, simple ovarian cyst, adenomyosis, breast nodule, polycystic ovaries, abdominal wall hernia, and simple breast cyst. It is important to note that of the 478 women, 97 (20.3%) had no findings, 115 (24.1%) had findings other than those summarized in Table 2, and 266 (55.6%) had at least one of the findings described in Table 2.

**Table 2:** Ultrasonographic findings in female inmates (N = 478).

Finding	Present n (%)	Absent n (%)
Uterine myoma	65 (13.6)	413 (86.4)
Biliary lithiasis	64 (13.4)	414 (86.6)
Renal lithiasis	55 (11.5)	423 (88.5)
Simple ovarian cyst	43 (9.0)	435 (91.0)
Adenomyosis	35 (7.3)	443 (92.7)
Breast nodule	30 (6.3)	448 (93.7)
Polycystic ovaries	26 (5.4)	452 (94.6)
Abdominal wall hernia	25 (5.2)	453 (94.8)
Simple breast cyst	20 (4.2)	458 (95.8)

Tables 3 to 7 show the correlation between ultrasonographic findings (uterine myoma, simple ovarian cyst, adenomyosis, breast nodule, and polycystic ovaries, respectively) and the sociodemographic characteristics of the female inmates. An ultrasonographic finding of uterine myoma was associated with older age (*p* = 0.022), higher BMI (*p* = 0.022) and being tattooed (*p* = 0.040). A finding of a simple ovarian cyst was associated with sexual orientation (*p* = 0.020), whereas adenomyosis were associated with older age (*p* = 0.012). An ultrasonographic finding of polycystic ovaries was associated with younger age (*p* < 0.001). The other ultrasonographic findings showed no statistical correlation with sociodemographic characteristics.

**Table 3:** Distribution of ultrasound findings of a uterine myoma according to the sociodemographic characteristics of female inmates.

Sociodemographic characteristic	Uterine myoma		*P*
	Present (n = 65)	Absent (n = 413)	
Age (years)			0.022^*^
Mean ± SD	42.1 ± 9.7	39.7 ± 10.6	
Median (range)	44 (23-71)	39 (22-73)	
Body mass index (kg/m^2^)			0.022^*^
Mean ± SD	26.94 ± 5.31	24.95 ± 4.83	
Median (range)	25.95 (17.99-	23.73 (16.53-	
	41.51)	49.59)	
Level of education, n (%)			0.173^†^
None	5 (35.7)	9 (64.3)	
< 9 years of schooling	26 (12.4)	183 (87.6)	
9 years of schooling	5 (10.2)	44 (89.8)	
High school, incomplete	9 (10.8)	74 (89.2)	
High school, complete	17 (16.8)	84 (83.2)	
College, incomplete	1 (7.1)	13 (92.9)	
College, complete	2 (25.0)	6 (75.0)	
Ethnicity, n (%)			0.480^†^
Asian	-	1 (100.0)	
White	28 (11.7)	212 (88.3)	
Black	10 (16.7)	50 (83.3)	
Mixed	27 (15.3)	150 (84.7)	
Tattooed, n (%)			0.040^†^
No	2 (4.1)	47 (95.9)	
Yes	63 (14.7)	366 (85.3)	
Sexual orientation, n (%)			0.991^‡^
Bisexual	14 (13.2)	92 (86.8)	
Heterosexual	39 (13.7)	246 (86.3)	
Homosexual	12 (13.8)	75 (86.2)	

* Mann-Whitney U test;

† Fisher’s exact test;

‡ Pearson’s chi-square test.

**Table 4:** Distribution of ultrasound findings of a simple ovarian cyst according to the sociodemographic characteristics of female inmates.

Sociodemographic characteristic	Simple ovarian cyst		*P*
	Present (n = 43)	Absent (n = 435)	
Age (years)			0.008^*^
Mean ± SD	37.4 ± 10.5	40.3 ± 10.5	
Median (range)	35 (23-71)	39 (22-73)	
Body mass index (kg/m^2^)			0.302^*^
Mean ± SD	24.3 ± 4.1	25.3 ± 5.0	
Median (range)	23.5 (18.2-	23.9 (16.5-	
	33.3)	49.6)	
Level of education, n (%)			0.303^*^
None	-	14 (100.0)	
< 9 years of schooling	14 (6.7)	195 (93.3)	
9 years of schooling	8 (16.3)	41 (83.7)	
High school, incomplete	10 (12.0)	73 (88.0)	
High school, complete	10 (9.9)	91 (90.1)	
College, incomplete	1 (7.1)	13 (92.9)	
College, complete	-	8 (100.0)	
Ethnicity, n (%)			0.549^*^
Asian	-	1 (100.0)	
White	23 (9.6)	217 (90.4)	
Black	7 (11.7)	53 (83.3)	
Mixed	13 (7.3)	164 (92.7)	
Tattooed, n (%)			> 0.999^*^
No	4 (8.2)	45 (91.8)	
Yes	39 (9.1)	390 (90.9)	
Sexual orientation, n (%)			0.020^‡^
Bisexual	5 (4.7)	101 (95.3)	
Heterosexual	24 (8.4)	261 (83.9)	
Homosexual	14 (6.1)	73 (90.8)	

* Mann-Whitney U test;

† Fisher’s exact test;

‡ Pearson’s chi-square test.

**Table 5:** Distribution of ultrasound findings of adenomyosis according to the sociodemographic characteristics of female inmates.

Sociodemographic characteristic	Adenomyosis		*P*
	Present (n = 35)	Absent (n = 443)	
Age (years)			0.012^*^
Mean ± SD	43.2 ± 7.4	39.8 ± 10.7	
Median (range)	43 (27-56)	39 (22-73)	
Body mass index (kg/m^2^)			0.304^*^
Mean ± SD	25.86 ± 4.56	25.17 ± 4.97	
Median (range)	24.44 (20.20-	23.88 (16.53-	
	37.46)	49.59)	
Level of education, n (%)			0.721+
None	-	14 (100.0)	
< 9 years of schooling	21 (10.0)	188 (90.0)	
9 years of schooling	3 (6.1)	46 (93.9)	
High school, incomplete	5 (6.0)	78 (94.0)	
High school, complete	6 (5.9)	95 (94.1)	
College, incomplete	-	14 (100.0)	
College, complete	-	8 (100.0)	
Ethnicity, n (%)			0.059^†^
Asian	1 (100.0)	-	
White	14 (5.8)	226 (94.2)	
Black	6 (10.0)	54 (90.0)	
Mixed	14 (7.9)	163 (92.1)	
Tattooed, n (%)			> 0.999+
No	3 (6.1)	46 (93.9)	
Yes	32 (7.5)	397 (92.5)	
Sexual orientation, n (%)			0.752^*^
Bisexual	7 (6.6)	99 (93.4)	
Heterosexual	20 (7.0)	265 (93.0)	
Homosexual	8 (9.2)	79 (90.8)	

* Mann-Whitney U test;

† Fisher’s exact test;

‡ Pearson’s chi-square test.

**Table 6:** Distribution of ultrasound findings of a breast nodule according to the sociodemographic characteristics of female inmates.

Sociodemographic characteristic	Breast nodule		*P*
	Present (n = 30)	Absent (n = 448)	
Age (years)			0.262^*^
Mean ± SD	43.1 ± 13.8	39.8 ± 10.2	
Median (range)	42.5 (22.0-70.0)	39.0 (22.0-73.0)	
Body mass index (kg/m^2^)			0.285^*^
Mean ± SD	24.14 ± 4.21	25.29 ± 4.98	
Median (range)	23.50 (17.26-	23.88 (16.53-	
	35.56)	49.59)	
Level of education, n (%)			0.182^†^
None	-	14 (100.0)	
< 9 years of schooling	12 (5.7)	197 (94.3)	
9 years of schooling	5 (10.2)	44 (89.8)	
High school, incomplete	4 (4.8)	79 (95.2)	
High school, complete	5 (5.0)	96 (95.0)	
College, incomplete	3 (21.4)	11 (78.6)	
College, complete	1 (12.5)	7 (87.5)	
Ethnicity, n (%)			0.606^*^
Asian	-	1 (100.0)	
White	18 (7.5)	222 (92.5)	
Black	3 (5.0)	57 (95.0)	
Mixed	9 (5.1)	168 (94.9)	
Tattooed, n (%)			0.218^*^
No	5 (10.2)	44 (89.8)	
Yes	25 (5.8)	404 (94.2)	
Sexual orientation, n (%)			0.687^‡^
Bisexual	6 (5.7)	100 (94.3)	
Heterosexual	20 (7.0)	265 (93.0)	
Homosexual	4 (4.6)	83 (95.4)	

* Mann-Whitney U test;

† Fisher’s exact test;

‡ Pearson’s chi-square test.

**Table 7:** Distribution of ultrasound findings of polycystic ovaries according to the sociodemographic characteristics of female inmates.

Sociodemographic characteristic	Polycystic ovaries		*P*
	Present (n = 26)	Absent (n = 452)	
Age (years)			< 0.001^*^
Mean ± SD	33.2 ± 5.6	40.4 ± 10.6	
Median (range)	34 (22-45)	40 (22-73)	
Body mass index (kg/m^2^)			0.639^*^
Mean ± SD	24.47 ± 3.84	25.26 ± 5.00	
Median (range)	23.80 (18.37-	23.88 (16.53-	
	35.16)	49.59)	
Level of education, n (%)			0.846^*^
None	-	14 (100.0)	
< 9 years of schooling	12 (5.7)	197 (94.3)	
9 years of schooling	2 (4.1)	47 (95.9)	
High school, incomplete	3 (3.6)	80 (96.4)	
High school, complete	8 (7.9)	93 (92.1)	
College, incomplete	1 (7.1)	13 (92.9)	
College, complete	-	8 (100.0)	
Ethnicity, n (%)			0.320^*^
Asian	-	1 (100.0)	
White	17 (7.1)	223 (92.9)	
Black	3 (5.0)	57 (95.0)	
Mixed	6 (3.4)	171 (96.6)	
Tattooed, n (%)			0.741^*^
No	3 (6.1)	46 (93.9)	
Yes	23 (5.4)	406 (94.6)	
Sexual orientation, n (%)			0.799^‡^
Bisexual	7 (6.6)	99 (93.4)	
Heterosexual	14 (4.9)	271 (95.1)	
Homosexual	5 (5.7)	82 (94.3)	

* Mann-Whitney U test;

† Fisher’s exact test;

‡ Pearson’s chi-square test.

**Figure 1:**
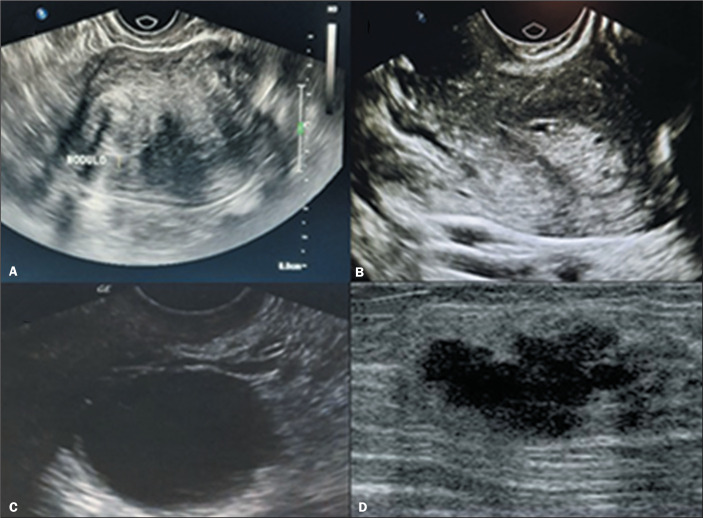
Ultrasonographic findings in female inmates. A: Uterine myoma. B: Uterine polyp. C: Simple ovarian cyst. D: Breast nodule suggestive of malignancy.

In the women with uterine myomas (Figure 1A), ultrasound examination allowed monitoring of the progression of menstrual symptoms, the identification of benign nodules, and the identification of uterine masses that were amenable to immediate surgical treatment. In six cases, submucosal myomas were diagnosed, with symptoms including excessive vaginal bleeding and clinical manifestations of anemia. In seven cases, the uterine volume was greater than 400 mL, being considerably greater in three cases (781, 1,000, and 1,400 mL, respectively). The women were all referred for surgery and passed the postoperative period, starting from postoperative day 3, in the prison, without any complications.

Of the 478 women evaluated, 35 (7.3%) had abnormal uterine bleeding and dysmenorrhea. In one of those women, the ultrasound image was characteristic of an endometrial cyst, and in another it was characteristic of an endometrioma. Because the most common complaints were abnormal uterine bleeding and dysmenorrhea, consistent with the suspicion of adenomyosis/endometriosis, and because ultrasound examination showed textural changes in the myometrium, and given the urgency of the cases, we routinely started treatment with contraceptives, preferably continuous, to relieve the symptoms. In all of those cases, the symptoms resolved without surgery. In three women with abnormal bleeding, ultrasound showed endometrial thickening. Those three women underwent surgical hysteroscopy, which revealed the presence of polyps (Figure 1B); after removal of the polyps, they had no more bleeding.

Among the female prison inmate population, the majority of ovarian ultrasound findings were simple cysts (in 7.7%), as depicted in Figure 1C, which could be promptly resolved with simple guidance on expectant management or, rarely, with the prescription of oral contraceptives.

Of the seven cases in which there were suspicious breast nodules, two were determined to be breast cancer: one was in the early stages and the other had metastasized to the cervical region. In the latter case (Figure 1D), the patient underwent surgery, radiotherapy, and chemotherapy, and remained stable until discharge.

## DISCUSSION

The focus of our study was to identify the most common endemic conditions among female inmates and to evaluate the impact of expediting ultrasound examinations for those waiting for the normal scheduling in the public health care system. The majority of our population consisted of Brazilian women, around 40 years of age, who were homemakers, were White, were high school graduates, had at least one tattoo, were evangelical Christians, were heterosexual, and were overweight. In a study involving 15 female prisons in eight Brazilian states, with a collective sample of 1,327 women, the population was overwhelmingly Black or mixed, of low socioeconomic status, poorly educated, and composed mainly of domestic workers^(10)^.

In the present study, relevant ultrasonographic findings were present in 60% of the female inmates evaluated, 40% of whom were symptomatic, which highlights the importance of valuing their complaints. In addition, ultrasound examination proved to be a valuable tool for diagnosing and monitoring health conditions, leading to more qualified care within the prison system. For 20% of the female inmates, ultrasound examination enabled the diagnosis of previously unknown conditions and prompt intervention in the more serious cases. In a study conducted in the American state of New Jersey, involving 908 female inmates, the prison appeared to improve access to behavioral health treatment among the women^(11)^. However, in the Australian state of Queensland, women prisoners were found to have poorer nutrition, exercise less, have higher rates of smoking, and be more likely to have asthma and diabetes, in comparison with women in the community^(12)^.

In our study, uterine disorders were the most prevalent, represented by uterine myomas (in 13.6%) and adenomyosis (in 7.3%). Transvaginal ultrasound has good sensitivity, specificity, and positive and negative predictive values for the diagnosis of adenomyosis in women scheduled for hysterectomy^(13)^. It has also been shown to be as effective as magnetic resonance imaging in detecting uterine myomas in premenopausal women who have undergone hysterectomy for benign reasons^(14)^. To our knowledge, there have been no previous assessing the rates of uterine disorders in female inmates.

With regard to breast disease, in any population, it is essential that precursor lesions and risk factors are always analyzed with a view toward early detection of breast cancer, which is the leading cause of cancer death among women in lowto middle-income countries and the second leading cause of such in high-income countries^(15)^. In our study, requests for the examination (by women with or without complaints) led to breast ultrasound being performed in 36.6% of the sample. Those examinations identified 31 nodules, seven of which were suspected to be malignant, and 24 cysts. Among the risk factors for breast cancer observed in our population, smoking was the most common. Other, less common, risk factors included obesity, a sedentary lifestyle, alcohol consumption, steroid use, a family history of breast cancer, and no history of breastfeeding^(16)^. In a cross-sectional survey including 100 incarcerated women in a unit prison in the American state of Rhode Island, 58% of the female inmates > 40 years of age reported having had a mammogram in the past two years^(17)^.

In our study sample, there were 14 cases of hepatic steatosis (mild in four and moderate in 10), which accounted for 2.5% of all ultrasonographic findings. In the general population of women, the reported prevalence of hepatic steatosis is 15-20% and there is evidence that metabolic dysfunction-associated steatotic liver disease is associated with insulin resistance, atherosclerosis, dyslipidemia, and arterial hypertension^(18)^. Despite the high prevalence of alcoholism among female inmates, our study showed that metabolic factors caused the most liver damage. The damage caused by alcohol depends on the frequency, the type of drink and constitutional differences^(19,20)^.

Biliary lithiasis was the second most prevalent ultrasonographic finding (in 13.4%). In the prison system, a sedentary lifestyle together with a diet rich in lipids and processed foods probably explains this finding, although there are other risk factors, such as age, female gender, elevated non-high-density lipoprotein cholesterol level, and a high BMI^(21,22)^. In a cross-sectional study of 1,013 female inmates in the state of São Paulo^(23)^, 47% were found to be overweight/obese and half were found to have high serum triglycerides. A high prevalence of daily consumption of ultra-processed foods was observed, with hot dog buns/sweet bread with margarine consumed by 86.5%, sugar-sweetened beverages by 68.4%, and cookies/candy by 77.1%.

In our study, renal lithiasis accounted for 11.5% of ultrasound findings. This condition is associated with comorbidities such as arterial hypertension, diabetes mellitus, obesity, liver disease, metabolic disorders, and low water intake. Hematuria and pain are the manifestations that lead people to seek emergency treatment^(24,25)^. The female inmates were given preventive measures for renal lithiasis based on lifestyle changes such as drinking 2.5-3.0 L of fluid per day, frequent urination, a diet rich in fiber and vegetables, controlled calcium intake (1.0-1.2 g/day), moderate sodium intake (4-5 g/day), moderate protein intake (0.8-1.0 g/day), and losing weight. As recommended in the literature^(24)^, the additional measures we prescribed included citrate supplementation and the administration of thiazide diuretics together with allopurinol (up to 300 mg/day).

In the vast majority of cases, ultrasound examination was sufficient to diagnose or confirm a pathology, contributing to prompt clinical or surgical treatment with good outcomes. Only in cases of suspected malignancy were women referred for other imaging modalities.

## CONCLUSION

The most common ultrasonographic findings in female inmates in a prison unit in the state of São Paulo, Brazil, were uterine myoma, biliary lithiasis, and renal lithiasis. This study opens up new perspectives for implementing policies that improve quality of life, as well as greater access to primary care for women in prisons.
